# Supplementary material for: How do midwives facilitate women to give birth during physiological second stage of labour? A systematic review
Source: PLoS One. 2020 Jul 28;15(7):e0226502. doi: 10.1371/journal.pone.0226502 (PMC7386622; doi:10.1371/journal.pone.0226502)
Supplement: S1 Table — (DOCX) [file pone.0226502.s002.docx]

**S2 Table. Search Strategy Tables**

**Search strategy in PubMed (5^th^ September 2019)**

| **#** | **Query** | **Results** |
| --- | --- | --- |
| **#6** | #5 NOT (animals[Mesh] NOT humans[Mesh]) | [**3567**](https://www.ncbi.nlm.nih.gov/pubmed/?cmd=HistorySearch&querykey=6) |
| **#5** | #1 OR #4 | [**3**](https://www.ncbi.nlm.nih.gov/pubmed/?cmd=HistorySearch&querykey=5)**652** |
| **#4** | #2 AND #3 | [**3232**](https://www.ncbi.nlm.nih.gov/pubmed/?cmd=HistorySearch&querykey=4) |
| **#3** | second stage*[tiab] | [**14758**](https://www.ncbi.nlm.nih.gov/pubmed/?cmd=HistorySearch&querykey=3) |
| **#2** | "Labor, Obstetric"[Mesh] OR "Parturition"[Mesh] OR "Delivery, Obstetric"[Mesh] OR labor[tiab] OR labour[tiab] OR birth*[tiab] OR childbirth*[tiab] OR parturition*[tiab] OR deliver*[tiab] | [**1010676**](https://www.ncbi.nlm.nih.gov/pubmed/?cmd=HistorySearch&querykey=2) |
| **#1** | "Labor Stage, Second"[Mesh] | [**1350**](https://www.ncbi.nlm.nih.gov/pubmed/?cmd=HistorySearch&querykey=1) |

**Search strategy in Embase (5^th^ September 2019)**

| **#** | **Query** | **Results** |
| --- | --- | --- |
| **#6** | #5 NOT ([animals]/lim NOT [humans]/lim) | **5037** |
| **#5** | #1 OR #4 | **5169** |
| **#4** | #2 AND #3 | **4625** |
| **#3** | 'second stage*':ti,ab | **18840** |
| **#2** | 'labor'/exp OR 'birth'/exp OR 'obstetric delivery'/exp OR labor:ti,ab OR labour:ti,ab OR birth*:ti,ab OR childbirth*:ti,ab OR parturition*:ti,ab OR deliver*:ti,ab | **1327847** |
| **#1** | 'labor stage 2'/exp | **2078** |

**Search strategy in CINAHL (5^th^ September 2019)**

| **#** | **Query** | **Results** |
| --- | --- | --- |
| **S6** | S5 NOT (MH "Animals" NOT MH "Human) | **1,569** |
| **S5** | S1 OR S4 | **1,569** |
| **S4** | S2 AND S3 | **1,300** |
| **S3** | TI "second stage*" OR AB "second stage*" | **2,766** |
| **S2** | ( (MH "Delivery, Obstetric+") OR (MH "Labor+") ) OR TI ( (labor OR labour OR birth* OR childbirth* OR parturition* OR deliver*) ) OR AB ( (labor OR labour OR birth* OR childbirth* OR parturition* OR deliver*) ) | **242,818** |
| **S1** | (MH "Labor Stage, Second") | **811** |

**Search strategy in PsycINFO (5^th^ September 2019)**

| **#** | **Query** | **Results** |
| --- | --- | --- |
| **S4** | S3 NOT (PO Animal NOT PO Human) | **141** |
| **S3** | S1 AND S2 | **146** |
| **S2** | TI "second stage*" OR AB "second stage*" | **1,858** |
| **S1** | ( (DE "Labor (Childbirth)" OR DE "Caesarean Birth" OR DE "Intrapartum Period"# OR #DE "Birth" OR DE "Caesarean Birth" OR DE "Natural Childbirth" OR DE "Premature Birth"# ) OR TI ( (labor OR labour OR birth* OR childbirth* OR parturition* OR deliver*) ) OR AB ( (labor OR labour OR birth* OR childbirth* OR parturition* OR deliver*) ) | **182,855** |

**Search strategy in the Cochrane Library (5^th^ September 2019)**

| **#** | **Query** | **Results** |
| --- | --- | --- |
| **#3** | #1 and #2 | **899** |
| **#2** | "second stage*":ti,ab,kw | **1668** |
| **#1** | (labor or labour or birth* or childbirt* or parturition or delivery):ti,ab,kw | **69026** |

**Search strategy in Maternity and Infant Care (5^th^ September 2019)**

| **#** | **Query** | **Results** |
| --- | --- | --- |
| **#10** | #1 or #9 | **1821** |
| **#9** | #7 and #8 | **1707** |
| **#8** | #2 or #3 or #4 or #5 or #6 | **115620** |
| **#7** | second stage.ti,ab. | **1760** |
| **#6** | (birth or childbirth or deliver*).mp. | **105765** |
| **#5** | labo?r.ti,ab. | **25410** |
| **#4** | Delivery.de. | **1344** |
| **#3** | parturition.ti,ab. | **566** |
| **#2** | Labour.de. | **16473** |
| **#1** | Labour stage - second.de. | **549** |
