# Supplementary material for: How do midwives facilitate women to give birth during physiological second stage of labour? A systematic review
Source: PLoS One. 2020 Jul 28;15(7):e0226502. doi: 10.1371/journal.pone.0226502 (PMC7386622; doi:10.1371/journal.pone.0226502)
Supplement: Attachment — Submitted filename: Response to Reviewers PONE-D-19-16084 .docx [file pone.0226502.s003.docx]

**Cover letter minor revision**

Christine E East,

Academic Editor

PLOS ONE

Dr Maria Healy

Lecturer in Midwifery (Education)

School of Nursing and Midwifery

Queen's University Belfast

97 Lisburn Road

Belfast, Northern Ireland

BT9 7BL

Telephone: +44 28 9097 2394

Email: [Maria.Healy@qub.ac.uk](mailto:Maria.Healy@qub.ac.uk)

PONE-D-19-16084

How do midwives facilitate women to give birth during physiological second stage of labour? A systematic review.

Maria Healy, Ph.D.; Viola Nyman, Ph.D.; Dale Spence, Ph.D; Rene H Otten; Corine Verhoeven, Ph.D.

Belfast, 17 October 2019

Dear Editor, Christine East,

We thank you and the reviewers for their comments on our manuscript entitled “How do midwives facilitate women to give birth during physiological second stage of labour? A systematic review” which we submitted for publication in PLOS ONE and for giving us the opportunity to revise the manuscript taking the comments into account.

We read the comments with interest and we adjusted the manuscript accordingly. We copied the comments of the reviewers and wrote our response in *Italics* underneath each comment. We included a revised manuscript with track changes and a clean version of the revised manuscript.

We hope these adjustments make the manuscript suitable for publication in your journal and we are looking forward to your reply.

Yours sincerely, on behalf of the co-authors,

Dr Maria Healy

Response to the comments of the editor and reviewers.

We note that the research questions of your systematic review are very broad, and the result and discussion consist of a narrative synthesis. In light of the methodological approach, used we feel that a scoping review may be a more suitable article type for your study. Therefore, we ask you if could please consider replacing the PRISMA checklist with the newly developed scoping review extension (PRISMA-ScR; available at <http://www.equator-network.org/reporting-guidelines/prisma-scr/>), making any relevant changes to the main manuscript.

*Thank you for this suggestion. However, we feel it would be appropriate to keep it as a systematic review. Our reasons for this are as following:*

*1.       The protocol has been published stating we are undertaking a systematic review*

*2.       All Systematic Review steps have been followed in detail*

*3.       Systematic reviews do frequently use a narrative synthesis – rather than a meta-analysis*

*4.      The questions were broad to capture a sufficient data base of studies to make it a meaningful systematic review.*

*However, we are willing to compromise to call our paper a systematic integrative review, if required by the editor.*

Please discuss the results of the quality assessment in the Results section

*These were added in the Results section, see page 8.*

Please update the search of your systematic review, including all new studies published since May 2018.
*Further to the editors request, we updated the search of our systematic review, including all new studies published since May 2018 until 5^th^ September 2019 and have adjusted the Results and the Discussion section accordingly.*

Please include captions for your Supporting Information files at the end of your manuscript, and update any in-text citations to match accordingly.

*We added captions for the Supporting Information files at the end of our manuscript.*

Reviewer #1:

The discussion part is the weakest part of your paper and I would suggest to work on this to get a stronger statement, you have begun in the introduction so strong and clear and this is in my opinion lost in the discussion.

*Thank you for this comment. We have further developed the Discussion section.*

Abstract
Here you are writing that you conducting "high quality intrapartum care" for the reader it would be helpful to read that it is about the second stage of childbirth.

*We added this in our revised manuscript.*

Introduction
Second sentence: I would suggest to cite a publication like this to have also a reference mentioned with physical effects of childbirth, even that your focus is another, but you have here a brought statement:
Keag OE, Norman JE, Stock SJ (2018) Long-term risks and benefits associated with cesarean delivery for mother, baby, and subsequent pregnancies: Systematic review and meta-analysis. PLoS Med 15(1): e1002494. https:// [doi.org/10.1371/journal.pmed.1002494](http://doi.org/10.1371/journal.pmed.1002494)

*Thank you for this suggestion, we added a sentence in the Introduction and included this reference.*

You write that the first stage of labour is characterised by regular "painful" contractions. Pain is something subjective. Not every woman is experiencing it as "painful" like woman who practicing hypnobirthing are coping quite well, in my experience. Please consider if you take the word out to have a more neutral definition.

*We do agree that women who practice hypnobirthing experience contractions in the first stage of labour not as painful however as most women will experience regular painful contractions, and as ‘painful’ is also stated in the WHO Intrapartum Care for a Positive Childbirth Experience Guideline first stage of labour definition (page 3); no adjustments were made in the revised manuscript.*

The definition/reference that you used for normal physiological birth is also saying that interventions should try to be avoided. You mention that normal physiological birth is associated with non-use of epidural, what is with other pharmacological pain-relief? As a reader I do not know how I should interpret your statement, here.

*We added ‘or other pharmacological pain relief’ to this sentence.*

I would also advice to write something about augmentation, because you exclude it in your search but in the definition, it is not clear that it is not part of a normal birth.

*We added a sentence about augmented and induced labour.*

I like the following section very much with the hormones!

*Thank you.*

What do you mean with "good quality intrapartum care" in your second RQ? Through your paper it is not getting clear. The other research question is addressed very well in the text!
*We removed this question since it is more or less the same question as our main research question, only phrased differently.*

Method
Fig. 2 is perfectly clear, but in the text it is not clear where you combined the AND for the second stage and the NOT for animals and humans.

*This information is provided in Supporting Information S2 Table. Search strategy tables*

The (S1 tables) is a bit lost here, and it would be helpful to be clearer what you mean.

*Here we meant Supporting Information S2 Table. Search strategy tables. We wrote this in revised manuscript.*

I can only guess what you want to say with the statement "reflecting the NICE intrapartum care guidance". I would love to read here your reasons for deciding on using NICE in an EU project, where a lot of other countries have also evidence-based guidelines that as you mentioned would be no problem to translate based on the COST members.

*The NICE Intrapartum care guidance is an internationally well-known and highly valued guideline, used or referred to in many countries.*

Results
You mention Barasinski et al, but there is no number, as a reader I must check the ref list to know if you already mentioned it as number 21 or 22. Can you please add the number?

*We apologize for this. We added the corresponding reference number in the manuscript.*

It would be helpful for the structure to have a sub-heading after the first paragraph before you go on to the remainder studies that you have synthesised in themes.

*We added the subheading “Themes” to the manuscript.*

p.11 on "Perineal massage" here it is not explicated if the massage is meant during pregnancy or in the second stage.

*Since our review focusses on what midwives can do to facilitate giving birth during the second stage of labour, perineal massage is meant during the second stage of labour. We clarified this in the revised manuscript.*

You write on p. 8 that the evidence-based aspect of midwifery practice during the second stage …. . I was wondering what is your comparison basis for "evidence-based" in this sentence. In your Method section, as I have read it, it is not defined. On page 12 in the discussion you make also a statement on the midwifery practice that "is not always evidence-based.".
*We changed “evidence-based into “consistent with the scientific literature”. See pages 8 and 12.*

Discussion
Here the first research question "how do midwives facilitate women to give birth during …" could be more discussed.

*Thank you for your comment. We further enhanced the Discussion section.*

I would have enjoyed to read more about the research gaps that you could not answer because of missing research in relation to your tow RQ. I do not find it enough to read it only in the conclusion. What are the next steps?

*We added a paragraph regarding research gaps and next steps.*

In the introduction you have cite the WHO 2018 on satisfaction, why do you mention this aspect not again in the discussion part?

*We mentioned the WHO 2018 in the Discussion section, as suggested by the reviewer.*

Reviewer #2:
I would suggest that you delete the subtitle in the Discussion section that is obsolete ("Principal Findings").

*Following the suggestion of the reviewer, we deleted the subtitle in the Discussion section. (page 14)*

In my opinion, "Discussion" section is the weakest part of your manuscript and needs some more work. I see a great deal of issues in the article that raise the question of professionalism of midwifery, autonomy, implementation of midwifery model of care in clinical practice etc. I think this would appeal international readers to read your article even more. Please consider this.

*Thank you for this addition. We rewrote the Discussion section.*
